# The Phenolic Compounds Profile and Cosmeceutical Significance of Two Kazakh Species of Onions: *Allium* *galanthum* and *A. turkestanicum*

**DOI:** 10.3390/molecules26185491

**Published:** 2021-09-09

**Authors:** Gulnara Kadyrbayeva, Justyna Zagórska, Agnieszka Grzegorczyk, Katarzyna Gaweł-Bęben, Marcelina Strzępek-Gomółka, Agnieszka Ludwiczuk, Karolina Czech, Manoj Kumar, Wojciech Koch, Anna Malm, Kazimierz Głowniak, Zuriyadda Sakipova, Wirginia Kukula-Koch

**Affiliations:** 1 School of Pharmacy, Kazakh National Medical University (KazNMU), Almaty 050012, Kazakhstan; chilnara_k@mail.ru (G.K.); sakipova.z@kaznmu.kz (Z.S.); 2 Chair and Department of Food and Nutrition, Medical University of Lublin, 20-093 Lublin, Poland; justyna.zagorska@umlub.pl (J.Z.); kochw@interia.pl (W.K.); 3 Chair and Department of Pharmaceutical Microbiology, Medical University of Lublin, 20-093 Lublin, Poland; agnieszka.grzegorczyk@umlub.pl (A.G.); anna.malm@umlub.pl (A.M.); 4 Department of Cosmetology, University of Information Technology and Management, 35-225 Rzeszów, Poland; kagawel@wsiz.edu.pl (K.G.-B.); mstrzepek@wsiz.edu.pl (M.S.-G.); kczech@wsiz.edu.pl (K.C.); kglowniak@wsiz.edu.pl (K.G.); 5 Independent Laboratory of Natural Products Chemistry, Department of Pharmacognosy, Medical University of Lublin, 20-093 Lublin, Poland; aludwiczuk@pharmacognosy.org; 6 Chemical and Biochemical Processing Division, ICAR-Central Institute for Research on Cotton Technology, Mumbai 400019, India; manoj.kumar13@icar.gov.in; 7 Chair and Department of Pharmacognosy, Medical University in Lublin, 20-093 Lublin, Poland

**Keywords:** *Allium*, Amaryllidaceae, antimicrobials, tyrosinase inhibition, HPLC-QTOF-MS/MS, GC-MS, antioxidants, polyphenols

## Abstract

Numerous species of *Allium* genus have been used in the traditional medicine based on their vast biological effects, e.g., antimicrobial, digestion stimulant, anti-sclerotic, soothing, antiradical or wound healing properties. In this work, unpolar and polar extracts from two lesser-investigated species of *Allium* growing in Kazakhstan, *Allium* *galanthum* Kar. & Kir. (AG) and *A. turkestanicum* Regel. (AT), were studied for their composition and biological effects. In the HPLC-ESI-QTOF-MS/MS analyses of water and alcoholic extracts simple organic acids, flavonoids and their glycosides were found to be the best represented group of secondary metabolites. On the other hand, in the GC-MS analysis diethyl ether, extracts were found to be rich sources of straight-chain hydrocarbons and their alcohols, fatty acids and sterols. The antimicrobial activity assessment showed a lower activity of polar extracts, however, the diethyl ether extract from AT bulbs and AG chives showed the strongest activity against *Bacillus subtilis* ATCC 6633, *B. cereus* ATCC 10876, some species of *Staphylococcus* (*S. aureus* ATCC 25923 and *S. epidermidis* ATCC 12228) and all tested *Candida* species (*Candida albicans* ATCC 2091, *Candida albicans* ATCC 10231, *Candida glabrata* ATCC 90030, *Candida krusei* ATCC 14243 and *Candida parapsilosis* ATCC 22019) with a minimum inhibitory concentration of 0.125–0.5 mg/mL. The highest antiradical capacity exhibited diethyl ether extracts from AG bulbs (IC50 = 19274.78 ± 92.11 mg Trolox eq/g of dried extract) in DPPH assay. In ABTS scavenging assay, the highest value of mg Trolox equivalents, 50.85 ± 2.90 was calculated for diethyl ether extract from AT bulbs. The same extract showed the highest inhibition of mushroom tyrosinase (82.65 ± 1.28% of enzyme activity), whereas AG bulb ether extract was the most efficient murine tyrosinase inhibitor (54% of the enzyme activity). The performed tests confirm possible cosmeceutical applications of these plants.

## 1. Introduction

The genus *Allium* L. is one of the largest among monocotyledons, as it includes over 800 species [1]. Over the years, the number of species belonging to the genus *Allium* has increased significantly.

The genus *Allium* is widespread throughout the Nearctic and Palearctic Region. The zone of its occurrence covers the area from the boreal zone to the dry areas of the subtropics [2]. Species belonging to this genus are found mainly in dry regions in the Northern Hemisphere. Its representatives are growing in Asia, North America, South Africa and Europe. However, it is Central Asia that is the most abundant area in various species belonging to the genus *Allium* and the main center of their distribution [3]. Interestingly, the flora of Kazakhstan is represented by 140 species of the genus, with 45 of them being endemic [4].

The herein presented study focuses on two insufficiently studied species of the described genus growing on the territory of Kazakhstan: *Allium* *Galanthum* Kar. & Kir. and *A. turkestanicum* Regel. The former also called snowdrop onion or *Allium* *pseudocepa* Schrenk [5] is growing in the areas from 500–1500 m above sea level, and forms clusters of bulbs, the diameter of which does not exceed 3 cm. The leaves of this plant resemble those of *Allium* *cepa* [6,7,8].

*A. turkestanicum* (AT)–a Turkestan onion, is a wild species native to Kazakhstan, Uzbekistan, Kyrgyzstan, Tajikistan and Turkmenistan [9], growing to an almost spherical grey bulb with a diameter of 1.5–3 mm. Its outer shells are paper-like, grey, without noticeable veins and the leaves are 2–10 mm wide, narrowed from the base to the top, and rough at the edge [10].

According to the Scopus database starting from 2009 the number of records related to the password ‘*Allium*’ exceeded 900 publications per year. A growing interest in the studies on *Allium* species encouraged the authors to undertake this topic, to determine the composition of extracts and to study the biological activity targeting their cosmeceutical applications of the selected two species of *Allium*.

The main active compounds of *Allium* species are sulphur containing metabolites and flavonoids. The former, that include cysteine sulphoxides are probably responsible for the characteristic taste and smell of plants belonging to the genus *Allium*. Alliin ((+)-*S*-(2-propenyl)-l-cysteine sulphoxide), isoalliin ((+)-*S*-(1-propenyl)-l-cysteine sulphoxide), propiine and methiine are the best described components the derivatives of cysteine sulphoxide [11,12,13]. Species belonging to the *Cepa* subgenus, including *A. galanthum*, are therefore characterized by a high concentration of the discussed compounds, which is related to their belonging to the third evolutionary line [14].

Traditional use of wild *Allium* species as natural remedies has been mostly due to a broad influence of sulphur compounds on the physiology of cells. Therefore, they are used as antibiotics, antimycotics and anti-cancer agents, especially in the treatment of gastric cancer [12,13,14,15]. Alliin and higher amounts of propiin have been tested to block the formation of nitrosamines in vitro, which reduces the risk of certain types of cancer. Moreover, alliin has been found to lower blood pressure [16].

Beside the above-described group of metabolites, polyphenols play an important role in the determination of the total biological activity of *Allium* extracts. Two groups of flavonoids, namely flavonols, the derivatives of quercetin, and anthocyanins, are the major representatives of phenolic components in the bulbs [17,18]. This particular group of secondary metabolites has a marked impact on the cosmetic significance of these plants. Previously 70% ethanolic extract from another species, *A. ursinum*, was proved to exhibit antioxidant and anti-tyrosinase properties and was claimed as a precious constituent suitable for the cosmetic and pharmaceutical industry [19]. Furthermore, an excellent work of Rocchetti and co-investigators [20] underlines the role of different *Allium* spp. in the inhibition of several types of enzymes, including tyrosinase that confirms cosmeceutical significance of some plant species from this genus. Phetmanee and colleagues [21] except from the aforementioned enzyme inhibitory effects underline the anti-melanogenic effect of *Allium* *ascalonicum* extract. Owing to a proved pharmacological potential of polyphenols that have been identified also in *Allium* spp. [18] and several reports on the cosmetic significance of the representatives of this genus, the Authors of this manuscript found it important to study the phenolic composition of *A. galanthum* (AG) and *A. turkestanicum* (AT) extracts and to focus on the bioactivity tests that may be important in terms of the future application in cosmetics. Having in mind a constant growth of cosmetic industry and a high value of cosmetics produced from natural resources, the study tends to find the possible applications of the two selected species for the production of plant-based cosmetics.

The survey involved compositional tests of the obtained extracts using HPLC-ESI-QTOF-MS/MS and GC-MS methods, and selected biological activity assays, namely the determination of antimicrobial, antioxidant and α-tyrosinase inhibitory properties to assess the pharmacological potential of the selected insufficiently studied onion species.

## 2. Results and Discussion

### 2.1. The Extracts Profiling by HPLC-ESI-QTOF-MS/MS

**Figure 1:**
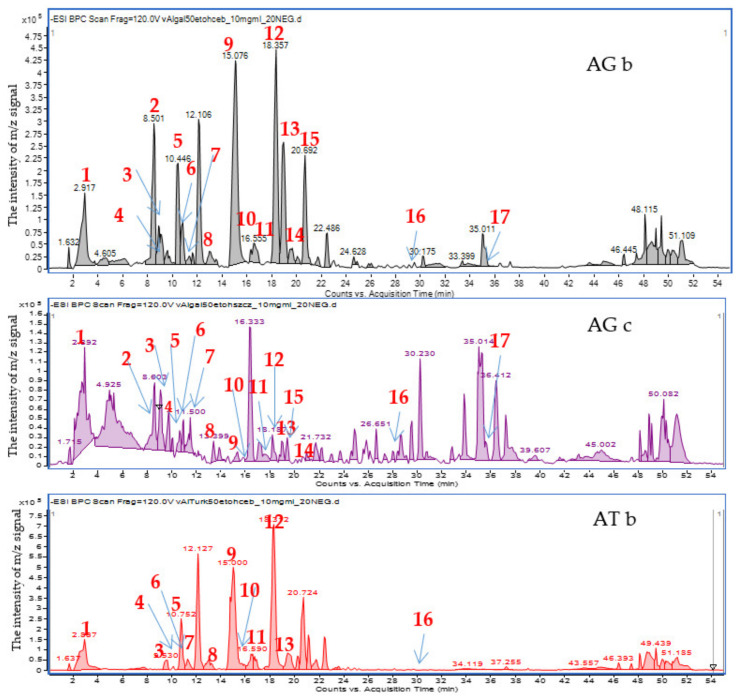
Mass chromatograms recorded in the negative ion mode for 50% ethanol extracts of Kazakhstani bulbs (top: *Allium* *galanthum* bulb extract (**AGb**), *Allium* *galanthum* chives extract (**AGc**), *Allium* *turkestanicum* bulb extract (**ATb**)).

The applied chromatographic conditions provided clear mass chromatograms with well-separated metabolites. Negative ionization mode was found preferable to the positive ion mode, as it provided higher sensitivity towards phenolic constituents of the tested extracts. The chromatograms presented in the Figure 1 and Figure S1 in the Supplementary File show the differentiation of the composition of onion (bulb) 50% ethanol extracts compared to the extracts obtained from the above-ground parts of plants (chives). Even if the composition of AG and AT bulb extracts is similar, the chromatogram of the latter species misses clear peaks at 8.5 and 35.5 min, and shows a less abundant one at 19.0 min.

The HPLC-ESI-QTOF-MS/MS analysis enabled a tentative identification of the major signals recorded in the obtained chromatograms. As it can be clearly seen, flavonoids and their glycosides constitute the major group of metabolites in the tested extracts. The list of tentatively identified compounds is presented in Table 1, and their MS/MS spectra are showed in the Table S1 in the Supplementary File.

The compositional studies revealed the presence of different types of flavonoids in the tested extracts. Kazakh onions were found to contain both flavonoids’ aglycones and glycosides the derivatives of quercetin, kaempferol, isorhamnetin, dihydroquercetin and an anthocyanin: cyanidin. However, the glycosylated forms were more abundant and in higher concentration from the aglycones. That was the case of quercetin, dihydroquercetin and kaempferol.

The performed chromatographic analyzes indicated that the composition of the bulb extract differed from the extracts obtained from the above-ground parts of plant. The bulbs also differ from each other in the content of individual compounds from the group of polyphenols, which could be seen in the differentiated intensities of the peak areas of the compounds of interest (see Table 1).

An interesting derivative of quercetin, namely its triglycoside, has been identified in the AG similarly to the results of the previously published study of Vijayalakshmi and co-investigators [26]. In the HPLC-MS analysis of the extract from AG chives, two signals for quercetin dihexoside were observed at the retention time of 10.6 and 12.6 min, contrary to the underground extracts’ constituents. The different two peaks can be derived from quercetin derivatives with a differently substituted sugar residue. Interestingly, in the case of the extracts from both bulbs, no second isomer of quercetin hexodise was observed. According to the obtained results, both compounds (*m*/*z* signal value 463 u) were present only in the above-ground parts of the plant. Recently Vijayalakshmi and collaborators [26] provided the tentative identification of the two isomers. Quercetin-7,4′-diglucoside was eluted as the first isomer in their analysis that was performed on a chromatographic column with a similar adsorbent (C18 silica gel), whereas quercetin-3,4′-diglucoside was identified as the second isomer.

The kaempferol content was the highest in AG chives. In turn, its amount in the AG bulbs was small, whereas in AT bulbs the presence of trace amounts was confirmed, similarly to its glycosides that were present only in small quantity. Thus, it can be concluded that AT differs in its profile from the other species where the presence of kaempferol was confirmed.

Kaempferol diglucoside that was confirmed in the studied extracts was present in two isoforms, and provided two peaks in the mass chromatograms at the Rt of 10.5 and 11.6 min. The two groups of signals may come from the two substitution isomers with the sugar moieties being attached at different sites to the kaempferol molecule. Previously other authors [23] found several isoforms of this flavonol in the extracts from Chinese chive juice (*Allium* *tuberosum*). In their HPLC-MS analysis these derivatives were called kaempferol diglucoside, or kaempferol sophoroside derivatives.

According to literature reports, kaempferol glucoside is the leading compound identified in extracts of the representatives of the genus *Allium* [30]. Its presence was also confirmed in the herein analyzed samples, although during the retention time of 17.5 min, the mass chromatograms also showed a smaller peak of the second kaempferol glucoside. Astragalin has a sugar attached at the 3-position to the aglycone backbone. In contrast, the second glucoside must differ in substitution to the main ring, but the scientific literature does not provide enough information to determine the second isomer. The 5- and 7-*O*-glycosides of kaempferol are found in nature, although it seems probable that the C-7 position of substitution is preferential for the representatives of *Allium* gender. 3,7-di-*O*-glucosides of kampferol were previously described in the scientific literature, e.g., in *Allium* *macrostemon* [30].

### 2.2. The GC-MS Identification of the Constituents of Diethyl Ether Extracts

Diethyl ether extracts obtained from bulbs and chives of *A. galanthum* and bulbs of *A. turkestanicum* were analyzed by use of GC-MS. The volatile components identified in the examined extracts are listed in Table 2 in order of their elution from ZB-5MS column. In total, 25, 22, and 17 compounds were identified in bulbs and chives of *A. galanthum* and bulbs of *A. turkestanicum*, respectively.

The most characteristic components present in all analyzed extracts are straight- and long-chain hydrocarbons and their alcohols. Hentriacontane with 31 carbons is the major component occurring in all three examined samples. Hentriacontane is a well-known alkane present in several plants of the family Euphorbiaceae (*Euphorbia*, *Alteurites*, *Colliguaja*) [31]. It also comprises about 10% beeswax [32]. Besides hentriacontane, the oxygenated long-chain saturated alkanes are characteristic for *A. galanthum* bulbs and chives. Bulbs produce mainly 1-octacosanol and 1-triacontalol, while chives 16-hentriacontanone (= palmitone) and 1-tritriacontanol. Another group of compounds found in these nonpolar extracts are fatty acids and their ethyl esters. Worth mentioning is the presence of linoleic acid only the bulbs of *A. turkestanicum*, while fatty acids ethyl esters in bulbs of *A. galanthum*. The next group of compounds identified in the analyzed extracts are sterols. All extracts contain 14-methylergost-8-en-3-ol, while in *A. galanthum* bulbs and chives cholesterol and an intermediate product of cholesterol, lathosterol. It seems very interesting to identify the cholesterol in *A. galanthum*, since there is a widespread belief that plants do not contain cholesterol–this is not true. Although the cholesterol concentration in plants is approximately several hundreds to thousands of times smaller than that of the animal tissues, it is not negligible [33,34]. It occurs as a compound building plant membranes and as a constituent of the surface lipids of leaves, where sometimes it may play a role of the major sterol [32,33,34].

### 2.3. The Determination of the Antimicrobial Activity of the Extracts

The best results of the antimicrobial activity test were obtained for the diethyl ether extracts contrary to the more polar water- and ethanol- containing ones. First, the study was performed for the bulb extracts only, although low MIC values that were obtained for the diethyl ether extracts encouraged the authors to check also the activity of AG chives. Table 3 compiles the results recorded for the diethyl ether extracts obtained from the bulbs and chives of AG and from the bulbs of AT.

As presented in Table 3, the diethyl ether extracts showed differential activity against tested reference bacteria (MIC = 0.125–4 mg/mL) and yeasts (MIC = 0.125–1 mg/mL). The ATb diethyl ether extract was characterized by a strong activity against bacteria (MIC = 0.125–1 mg/mL) and yeasts (MIC = 0.125–0.5 mg/mL). In contrast, a lower antimicrobial activity of the AGb diethyl ether extract (MIC = 0.25–4 mg/mL) was showed. However, the diethyl ether extract obtained from AGc was characterized by the higher activity and the lowest MIC values (MIC = 0.125–1 mg/mL), similar to ATb diethyl ether extract. All Gram-positive bacteria and yeasts were sensitive to diethyl ether extracts from onions of *A. turkestanicum* with MIC = 0.125–1 mg/mL and MIC = 0.125–0.5 mg/mL, respectively. The tested Gram-negative bacteria showed good sensitivity to the diethyl ether extracts from ATb and AGc at concentration 1 mg/mL.

These extracts exhibited strong effect towards *Bacillus* spp. ATCC (MIC = 0.125–0.25 mg/mL) and *Staphylococcus aureus* ATCC 25923 (MIC = 0.5–1 mg/mL). Moreover, these extracts possessed strong properties against all tested *Candida* strains ATCC (MIC = 0.125–0.5 mg/mL).

In addition, water extracts and ethanol extracts (EtOH50%, EtOH70% and EtOH96%) obtained from AGb and ATb were also investigated for their antimicrobial properties (see Table 4 and Table 5). This study concluded that water extracts obtained from AG showed a weaker antimicrobial activity (for bacteria: MIC = 16–32 mg/mL and for yeasts: MIC = 8 mg/mL) compared to water extracts from AT (for bacteria: MIC = 4–16 mg/mL and for yeasts: MIC = 1–2 mg/mL) (Table 4 and Table 5).

The water extract and the 50% ethanol extract from AG had the same activity against bacteria (MIC = 16–32 mg/mL) and yeast (MIC = 8 mg/mL) (Table 4), in contrast to the AT extracts, where the 50% ethanol extract (EtOH50%) was more active than the water extract (Table 5). The activity of all ethanol extracts (EtOH50%, EtOH70%, EtOH96%) from both species against yeast did not differ much. Their MIC values were calculated as 8 mg/mL for AG and 0.5–1 mg/mL for AT. The EtOH70% and EtOH96% extracts did not differ in activity within the same bulb species. The ethanol extracts of AT showed 4–16 times better activity than the ethanol extracts from AGb on the basis of MIC values.

The MIC values calculated for the reference antimicrobial substances were as follows: MIC of vancomycin for *S. aureus* ATCC 29213 was 1 µg/mL, MIC of ciprofloxacin for *Escherichia coli* ATCC 25922 was 0.015 µg/mL and MIC of fluconazole for *Candida albicans* ATCC was 1 µg/mL.

According to the data presented in Table 3, Table 4 and Table 5, most of the extracts possessed bactericidal (MBC/MIC = 1–4) and fungicidal effect (MFC/MIC = 1–4). The antimicrobials are generally regarded as bactericidal or fungicidal agents if the MBC/MIC or MFC/MIC ratio is ≤4, and it is agreed that antimicrobials are usually regarded as bacteriostatic or fungistatic if the MBC/MIC or MFC/MIC ratio is >4 [35]. The bacteriostatic effect was noted with some diethyl ether extracts against some Gram-positive and some Gram-negative bacteria (MBC/MIC = 8).

In summary, in comparison with diethyl ether extracts, a lower antimicrobial activity of all ethanol and water extracts was shown by both AT and AG. 

The highest microbiological activity of diethyl ether extracts from bulbs of *A. turkestanicum* was observed against *B. subtilis* ATCC 6633, *B. cereus* ATCC 10876, *S. aureus* ATCC 25923, *S. epidermidis* ATCC 12228 and all tested *Candida* species: *C. albicans* ATCC 2091, *C. albicans* ATCC 10231, *C. glabrata* ATCC 90030, *C. krusei* ATCC 14243 and *C. parapsilosis* ATCC 22019 (MIC = 0.125–0.5 mg/mL) as showed in the tables. High antifungal properties of diethyl ether extracts from AGb and ATb were also proved.

The obtained results indicate the possibility of various health benefits and practical use of diethyl ether extracts obtained from ATb and AGc in preparations for external use active against gram-positive bacteria, mainly *S. aureus* and in preparations for internal use in food poisoning caused by *B. cereus*, as well as against fungal infections mainly caused by *Candida* spp.

The observed higher activity of the diethyl ether extracts may be explained by the results elaborated during the GC-MS analysis. The studied samples contained the oxygenated long-chain saturated alkanes. Among them, 16-hentriacontanone (= palmitone) and 1-tritriacontanol were identified. Those metabolites were previously reported as active antimicrobial agents. The former compound was isolated from the leaf cuticular waxes obtained from *Annona squamosal* by Shiva Shanker and collaborators [36] as the major component. Their studies showed a higher antimicrobial potential of palmitone from other constituents of the wax. It is worth a note that *Bacillus* species tested in their study were especially sensitive to this component (MIC = 6.25 µg/mL), which is in accordance with the results of the herein presented assay. Furthermore, long chain primary alcohols, like 1-triacontanol and heptadecanol were previously proved to influence the antimicrobial properties of *Solena amplexicaulis* leaves, which was described by Chatterjee and co-investigators [37].

These constituents that were present in the unpolar extract could determine the total activity of the samples. Similarly, in the studies of Tomovic and co-investigators [38], the chloroform extract from *Allium* *ursinum* chives exhibited a significantly stronger antimicrobial action than methanol or water extracts. Chloroform extract from the chives was the most active against *B. subtilis* ATCC 6633 (MIC = 0.313 mg/mL), for *B. cereus* (MIC = 2.5 mg/mL) and *S. aureus* (MIC = 2.5 mg/mL).

The herein presented data show a marked antimicrobial potential of the studied extracts. Previously, Santas et al. [39] published the results of antimicrobial assay performed on three varieties of *Allium* *cepa* growing in Spain. Ethyl acetate extracts from the onions were stronger from water extracts, but the range of calculated MIC values was still 40− > 100 mg/mL.

Four years later, Bakht et al. [40] showed a similar activity ethanol extracts of *A. cepa* which did not inhibit the growth of *B. subtilus*, *S. aureus*, *P. aeruginosa* and *S. typhi* at any concentrations. At a concentration 2 mg/disc, the water extracts were effective in inhibiting the growth of *B. subtilus* and *K. pneumoniae*. Ethanol extracts were not active against *E. coli*, and they inhibited the growth of *C. albicans* only at concentrations >1 mg/disc. Similar results were also shown by Hughes and Lawson [41], Bekenblia [42] and Chathradhyunthi et al. [43].

The activity of AT and AG extracts was also higher from those reported by Fredotovic et al. [44] for *A.* *x* *cornutum* and *A. cepa* (yellow and red variety) onion peel extract. Their recent publication presents the results of antimicrobial assay that was performed on the waste extracts from these two species. The reported MIC values for *S. aureus* were within the range of 7.8–500 mg/mL, for *B. cereus:* 125–500 mg/mL, for *E. coli:* 500–2000 mg/mL, for *K. pneumoniae*: 500− > 2000 mg/mL, and for *C. albicans*: 1000− > 2000 mg/mL.

### 2.4. The Assessment of the Antioxidant Activity of A. galanthum and A. turkestanicum Extracts

Extracts from bulbs, chieves and flowers of several *Allium* species are known for their significant antioxidant activity. Strong antioxidant potential was found to be related with sulfur-containing compounds and flavonoids [45].

The relationship between *A. galanthum* and *A. turkestanicum* extracts concentration and the percentage of neutralized DPPH and ABTS radicals is shown in the Supplementary Figures S2 and S3. Table 6 presents the antioxidant potential of the analyzed extracts displayed as Trolox equivalents per gram of dried extract weight. Interestingly, the most significant antioxidant activity in the DPPH scavenging assay was detected for diethyl ether extracts from *A. galanthum* bulbs (AGb_Eth), while diethyl ether extract from *A. turkestanicum* bulbs (ATb_Eth) was the most active in ABTS scavenging assay. Ethanol and water extracts were found to exhibit a weaker antioxidant potential.

The analysis of antioxidant properties of four *Allium* species grown in Italy, *A. neapolitanum*, *A. roseum*, *A. sativum* and *A. subhirsutum*, showed that the extracts from chives (leaves) and flowers possess higher antioxidant activity than bulb extracts. The antioxidant activity of *Allium* extracts directly correlated with the total polyphenolics content [46]. Similar observation was made for *A. ursinum* extracts: the phenolic content and antioxidant potential measured using DPPH, ABTS and FRAP assays was also higher in chives than in bulb extracts [47].

Additionally, a higher antioxidant activity of unpolar chloroform *Allium* extracts, in comparison with aqueous or alcoholic extracts, was shown previously, for example for *A. ursinum* leaves [41].

The predominant components identified in AGb_Eth extract were aliphatic alcohols, which are more likely responsible for the superior antioxidant activity of this extract. Comparative analysis of milk thistle (*Silybium marianum* L.) seed oil showed that higher content of aliphatic alcohols in immature oil results in more significant antioxidant activity in DPPH and ABTS scavenging assays when compared with matured oil, containing about 30% less of these compounds. Triacontanol and dotriacontanol were major components of strongly antioxidant immature milk thistle oil [48]. These alcohols were also identified among predominant components of AGb_Eth extract.

Antioxidant activity of ATb_Eth extract might result from the presence of linoleic acid, which was not identified in AGb and AGc extracts. Plant oils, such as cacay oil, containing high amounts of linoleic acid (58.3%), were previously shown to display stronger antioxidant activity than cacay butter and coconut oil, with low linoleic acid content (16.8% and 6.1%, respectively) [49].

### 2.5. Tyrosinase Inhibition by A. galanthum and A. turkestanicum Extracts

Tyrosinase (EC. 1.14.18.1) is a key enzyme of melanogenesis, catalyzing the conversion of l-tyrosine to l-dihydroxyphenylalanine (l-DOPA) and subsequently to dopaquinone. Several natural extracts and plant-derived compounds have been shown in previous years as effective tyrosinase inhibitors, and are used as active ingredients in cosmetics and medical ointments [50]. Mushroom tyrosinase inhibitory assay is the most widely used method to search for new tyrosinase inhibitors. However, due to the structural and functional differences between mushroom and mammalian enzymes, mushroom tyrosinase inhibitors are not always effective against mammalian tyrosinase [51]. For example, significant differences between mushroom and tyrosinase inhibitory activity were reported for *Achillea biebersteinii* extracts and isolated compounds [52].

**Figure 2:**
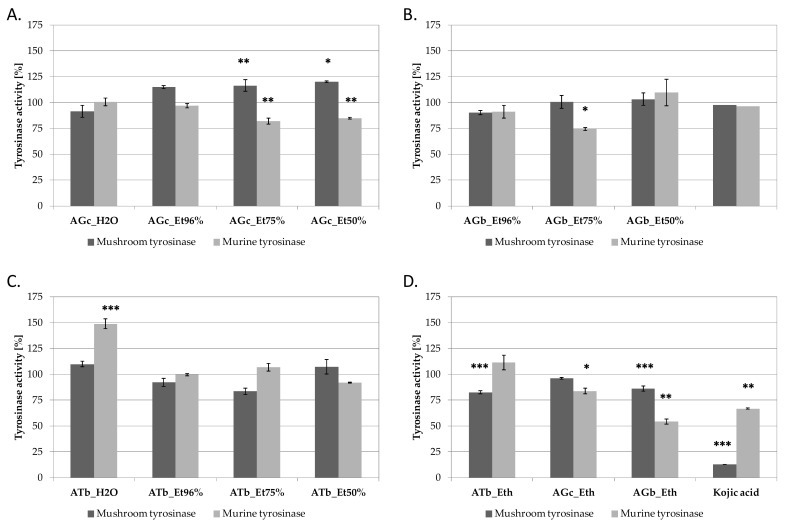
Mushroom and murine tyrosinase inhibitory activity of *A. galanthus* chives (**A**) and bulbs (**B**) (AGc and AGb, respectively), *A. turkestanicum* bulbs (ATb) (**C**) and diethyl ether extracts from AGc, AGb and ATb (**D**) extracts at 100 µg/mL in comparison with 100 g/mL kojic acid; values on graphs represent mean ± SD (*n = 3*), *** *p* < 0.001, ** *p* < 0.01, * *p* < 0.05.

AG and AT extracts were compared for their tyrosinase inhibtory activity using commercially available mushroom tyrosinase and B16F10 cell lysate, containing murine tyrosinase. Kojic acid was used in both assays as inhibitory control (Figure 2). Among *A. galanthum* chives extracts, extracts AGc_Et50% and AGc_75% were found to significantly inhibit murine tyrosinase and slightly activate mushroom enzyme (Figure 2A). Aqueous and ethanolic extracts from *A. galanthum* bulbs were not influencing tyrosinases activity, except of AGb_Et96% extract inhibiting mushroom tyrosinase by ca. 25% at 100 µg/mL (Figure 2B). The most active murine tyrosinase inhibitors were found in AGb_Eth extract, decreasing the activity of murine tyrosinase by 46% at 100 µg/mL (Figure 2D). Interestingly, aqueous extract from *A. turkestanicum* bulbs (ATb_H_2_O) increased the activity of murine tyrosinase (Figure 2C).

Tyrosinase inhibitory potential was previously described for other *Allium* species. *A. nigrum* L., and *A. subhirsutum* methanolic extracts from bulbs and aerial parts were found to inhibit mushroom tyrosinase [53]. Tyrosinase inhibitory activity was also shown for *Allium* *scorodoprasum* L. subsp. *rotundum*, but in this species the highest tyrosinase inhibitory potential was found for the flower extract [54].

Quercetin 4′-*O*-β-*D*-glucopyranoside and quercetin-3’-*O*-beta-*D*-glucoside are the two mushroom tyrosinase inhibitors isolated from methanolc extracts of *A. cepa* dried skin. The IC_50_ values for the first compound were 4.3 and 52.7 µM in assays with l-tyrosine or l-DOPA substrates, respectively. Quercetin-3’-*O*-beta-*D*-glucoside inhibited mushroom tyrosinase with an IC_50_ value of 6.5 µM using l-tyrosine and 48.5 µM using l-DOPA as substrates, respectively [55]. Quercetin-3’-*O*-beta-*D*-glucoside was also found to downregulate melanin biosynthesis in B16F10 melanoma cells with an IC_50_ value of 38.8 µM [55].

(*S*)-*N*-trans-feruloyloctopamine isolated from *A. sativum* skin was found as effectve mushroom tyrosinase inhibitor and decreased the relative melanin contents in a dose-dependent manner in the α-MSH-stimulated B16F10 cells. Molecular analysis revealed that this compound inhibits melanogenesis by down-regulating tyrosinase mRNA and protein expression levels [56].

## 3. Materials and Methods

### 3.1. Chemicals and Reagents

Reagent grade absolute ethyl alcohol and ether used for the preparation of extracts, were purchased from Avantor Performance Materials (Gliwice, Poland). The solvents for HPLC-MS analyses (water, 0.1% formic acid and acetonitrile) were manufactured by J.T. Baker (Phillipsburg, NJ, USA).

Dulbecco’s modified Eagle’s medium (DMEM)/high glucose, Dulbecco’s phosphate buffered saline (DPBS), *Agaricus bisporus*, mushroom tyrosinase 3,4-dihydroxy-l-phenylalanine (l-DOPA), 2,2-diphenyl-1-picrylhydrazyl (DPPH), 2,20-azino-bis (3-ethylbenzothiazoline-6-sulfonic acid (ABTS), Trolox, Triton X-100 and kojic acid were purchased from Sigma Aldrich (St. Louis, MO, USA). K_2_S_2_O_8_ was obtained from Chempur (Piekary Slaskie, Poland). Fetal bovine serum (FBS) was obtained from Pan-Biotech (Aidenbach, Germany). B16F10 murine melanoma cell line (ATCC CRL-6475) was purchased from LGC Standards (Łomianki, Poland).

### 3.2. Plant Material

Plant material investigated in the present study represented the bulbs and chives of AG and the bulbs of AT. Both species were collected on the outskirts of Almaty (Kazakhstan). The collection of *A. turkestanicum*, growing on loamy soil, was carried out in clear sunny weather by hand, avoiding the ingress of foreign parts of the plant. Coordinates of the collection area near the village of Targap, Almaty region: W 43.29320031385285, E 75.50903320312501, at an altitude of 763 m above sea level. Collection and harvesting of wild raw materials of *A. galanthum* Kar. et Kir. in the phase of leaf formation in the mountainous part of the elevated valley to the north-west of the city of Almaty in the Kogaly tract of the Anrakai upland at an altitude of 1023 meters above sea level. The collected material was identified in the Department of Pharmacy at Kazakh National Medical University by Doctor Zuriyadda Sakipova and brought to the Department of Pharmacognosy of the Medical University of Lublin as fresh. The extracts were obtained immediately according to the protocol described below.

### 3.3. Extraction

Bulbs and green shoots were finely chopped and extracted using absolute ethanol, 70% ethanol, 50% ethanol and water by ultrasound-assisted maceration at room temperature for 30 minutes in three cycles, using a fresh portion of solvent every time (solid to liquid ratio: 1:10 *w*/*v*). The same extracts were joined, concentrated using a rotary evaporator to dryness at a temperature not exceeding 45 °C, and kept in the freezer at −20 °C before the bioactivity and compositional studies. Prior to HPLC-MS analyses, the extracts were resuspeded in methanol to a concentration of 10 mg/mL and filtered through a syringe filter with a pore size of 0.22 µm (CronusFilters, Camberley, UK). The diethyl ether extracts were obtained by crushing the finely cut material with a pistle in a mortar together with the solvent for 5 min. The obtained extract was passed through a small glass column filled with silica gel (NP silica gel 60, 0.063–0.200, Merck, Darmstadt, Germany) bed to remove water, and left opened in a weighted vial for evaporation. Dried residue was used for biological studies, while for the GC-MS based compositional study, the dry extract was re-dissolved in diethyl ether to the concentration of 20 mg/mL.

### 3.4. HPLC-ESI-QTOF-MS/MS Analysis of Extracts from Bulbs and Chives of A. galanthum and Bulbs of A. turkestanicum

The chromatographic analysis was performed using an HPLC-ESI-Q-TOF-MS platform composed of a mass spectrometer (6500 Series Agilent Technologies, Santa Clara, CA, USA) and an HPLC system (1200 Series, Agilent Technologies, Santa Clara, CA, USA) and using Zorbax Eclipse Plus RP 18 column (150 mm × 2.1 mm, d_p_ = 3.5 µm) manufactured by Agilent Technologies. In details, the set was equipped with a binary pump (G1312C), a degasser (G1322A), a PDA detector (G1315D) and an autosampler (G1329B). Specific parameters of chromatographic determinations, were as follows: the flow rate 0.2 mL/min, the thermostat temperature 20 °C, the injection volume 20 µL, the UV detection range of 190–600 nm, the DAD detection at 280 and 320 nm, the run time of 55 min, the composition of the mobile phase: solvent A, 0.1% aqueous solution of formic acid, solvent B, 0.1% of formic acid in acetonitrile; the gradient of B: 0 min 1% B, 2 min 15% B, 40 min 40% B, 45 min 95% B, 47 min 1% B.

Mass spectra that were recorded in both positive and negative ionization modes and within the *m*/*z* range of 50–1200 Da were obtained using the following mass spectrometer settings: the capillary voltage of 3.5 kV, gas flows of 12 L/min, voltage of 120 V, gas and sheath gas temperatures of 350 and 325 °C, respectively, fragmentation skimmer voltage of 65 V and the collision energies of 10 and 20 V. In the prepared method, the MS/MS fragmentation spectra were obtained for the two most intense *m*/*z* signals in every scan. After the collection of one MS/MS spectrum, the selected *m*/*z* signals were later excluded for the following 0.2 min from re-fragmenting. Thanks to the applied conditions, the less-intensive signals could be also fragmented. The identification of the components in the extracts was based on their fragmentation spectra, in a direct comparison with the solutions of standards, based on the analysis literature data and open databases (Metlin), and on the recorded retention times.

### 3.5. The GC-MS Analysis of Diethyl Ether Extracts

The GC–MS analysis of volatiles present in extracts was performed with a Shimadzu GC-2010 Plus GC instrument coupled with a Shimadzu QP2010 Ultra mass spectrometer (Kyoto, Japan). The analytes were separated on a ZB-5 MS capilary column (30 m, 0.25 mm i.d.) with a film thickness of 0.25 m (Phenomenex, Torrrance, CA, USA). The oven temperature program was as follows: 50 °C initially for 3 min, increased to 250 °C at 5 °C/min, and held for 5 min; and finally the temperature was increased to 320 °C at the rate of 10 °C/min and held for 5 min. The total time of analysis was 60 min. Helium was used as a carrier gas of 1.0 mL/min flow rate. A split injection was performed with a split ratio of 1:20. The injector port, interface and ion source temperatures were kept at 280, 250 and 220 °C, respectively. The spectrometer was operated in the electron impact mode, the scan range was 40–600 amu, the ionization energy was 70 eV and the scan rate was 0.20 s per scan. The retention indices were determined by analyzing a solution containing the homologous series of n-alkanes (C8–C34) under the same operating conditions. Compounds were identified using computer-assisted spectral libraries (MassFinder 2.1; NIST 2011).

### 3.6. The Antibacterial Assay In Vitro

Minimum inhibitory concentration (MIC) of the extracts obtained from *Allium* *galathum* and *A. turkestanicum* for seventeen reference strains American Type Culture Collection (ATCC), including seven Gram-positive bacteria (*Staphylococcus epidermidis* ATCC 12228, *S. aureus* ATCC 25923, *S. aureus* ATCC 29213, *S. aureus* ATCC BAA 1707, *B. subtilis* ATCC 6633, *B. cereus* ATCC 10876, *M. luteus* ATCC 10240) and five Gram-negative bacteria (*E. coli* ATCC 25922, *S. typhimurium* ATCC 14028, *K. pneumoniae* ATCC 13883, *B. bronchiseptica* ATCC 4617, *P. mirabilis* ATCC 12453) and five yeasts (*C. albicans* ATCC 2091, *C. albicans* ATCC 10231, *C. glabrata* ATCC 90030, *C. krusei* ATCC 14243, *C. parapsilosis* ATCC 22019) was performed by the microdilution broth method according to the European Committee on Antimicrobial Susceptibility Testing (EUCAST) [35]. Minimal bactericidal concentration (MBC) or minimal fungicidal concentration (MFC) was also determined. The antimicrobial studies were performed as described previously [57]. Briefly, the stock solutions of extracts were prepared in a diluted solution of dimethyl sulphoxide (DMSO) that did not exceed 2% in the final mixture.

Each experiment was repeated in triplicate. The most common representative data were presented of the three MIC and MBC or MFC values, i.e., the mode.

The standard chemotherapeutics agents: vancomycin (Va; range of 0.06–16 μg/mL), ciprofloxacin (CIP; range of 0.015–16 μg/mL), and fluconazole (FLU; range of 0.06–16 μg/mL were used as antimicrobial substances active against Gram-positive bacteria, Gram-negative bacteria and yeasts.

### 3.7. The DPPH Scavenging Assay

DPPH radical scavenging assay was performed according to the procedure described by Matejic et al. [58], with modifications. 50 μL of appropriately diluted extract (1.2 mg/mL, 0.6 mg/mL, 0.3 mg/mL, 0.15 mg/mL, 0.075 mg/mL, 0.0375 mg/mL, 0.01875 mg/mL, 0.00938 mg/mL, 0.00469 mg/mL, 0.00234 mg/mL, 0.00117 mg/mL and 0.00059 mg/mL) were mixed with 50 μL DPPH working solution (25 mM in 99.9% methanol; A_540_ ≈ 1). 50 μL of solvent mixed with 50 μL DPPH served as a control sample. Following 10 min incubation at room temperature in darkness, the absorbance of the samples was measured at λ = 540 nm using FilterMax F5 microplate reader (Molecular Devices, San Jose, CA, USA). The measured values of measurements were corrected by the absorbance value of the sample without DPPH. The percentage of DPPH radical neutralization was calculated using the following Equation

% of DPPH˙ scavenging = (1−(Abs(S)/Abs(C))) × 100(1)

where Abs(S)-the corrected absorbance of the extract, Abs(C)-the corrected absorbance of the control sample (DPPH + solvent).

The analysis was conducted in three independent repetitions, using vitamin C as a reference compound. The calibration curve (y = −0.1241x + 0.24; R^2^ = 0.9974) was prepared using 0–1 mg/mL trolox. The content of total antioxidants in each sample was calculated as miligrams of trolox equivalents per gram of dried extract weight (TE/g dw).

### 3.8. ABTS Radical Scavenging Assay

ABTS radical scavenging assay was preformed according to the protocol described by Re and co-workers [59] with modifications. ABTS working solution was prepared by mixing 7 mM ABTS in 2.45 mM K_2_S_2_O_8_ with distilled H_2_O in order to obtain A_405_ ≈ 1.15 μL of diluted extracts (1.2 mg/mL, 0.6 mg/mL, 0.3 mg/mL, 0.15 mg/mL, 0.075 mg/mL, 0.0375 mg/mL, 0.01875 mg/mL, 0.00938 mg/mL, 0.00469 mg/mL, 0.00234 mg/mL, 0.00117 mg/mL and 0.00059 mg/mL) were mixed with 135 μL ABTS working solution. 15 μL of solvents mixed with 135 μL ABTS were used as control samples. The absorbance of the samples was measured at λ = 405 nm using FilterMax F5 microplate reader (Molecular Devices, San Jose, CA, USA) following 15 min of incubation at room temperature in darkness. The measured values were corrected by the absorbance value of the sample without ABTS. The percentage of ABTS radical scavenging was calculated based on the following Equation

% of ABTS scavenging = (1 − (Abs(S)/Abs(C))) × 100(2)

where Abs(S)—the corrected absorbance of the extract, and Abs(C)_—_the corrected absorbance of the control sample (ABTS + solvent).

The analysis was conducted in three independent repetitions, using vitamin C as a reference compound. The calibration curve (y = −187.01x + 0.3589; R^2^ = 0.9992) was prepared using 0–1 mg/mL trolox. The content of total antioxidants in each sample was calculated as miligrams of trolox equivalents per gram of dried extract weight (TE/g dw).

### 3.9. Mushroom Tyrosinase Inhibitory Assay

In order to assess the inhibition of the monophenolase activity of mushroom tyrosinase by *Allium* sp. extracts, the protocol described by Wang and co-workers was applied [60]. 20 µL of diluted sample was mixed with 80 µL of phosphate buffer (100 mM, pH = 6.8) was mixed with 80 µL of phosphate buffer (100 mM, pH = 6.8) and 20 µL mushroom tyrosinase working solution (500 U). The samples were pre-incubated for 10 min at room temperature, 80 µL 2 mM L-tyrosine was added and the samples were incubated for further 20 min at room temperature, in darkness. The method described by Uchida et al. [61] was implicated to analyze the inhibition of the diphenolase activity of mushroom tyrosinase. 20 µL of diluted sample was mixed with 120 µL of phosphate buffer (100 mM, pH = 6.8) and 20 µL tyrosinase solution (500 U). The samples were pre-incubated for 10 min at room temperature, then 40 µL 4 mM l-DOPA was added and the samples were incubated for a further 20 min at room temperature, in darkness. In both analyses the absorbance of formed dopachrome was measured using FilterMax F5 microplate reader (Molecular Devices, San Jose, CA, USA) at λ = 450 nm. The obtained values were corrected by the absorbance of the extracts without tyrosinase and the substrate (L-tyrosine or l-DOPA). Control sample (100% tyrosinase activity) was composed of phosphate buffer, tyrosinase, substrate and equal volume of the solvent. 100 µg/mL kojic acid was used as a reference tyrosinase inhibitor. Tyrosinase was calculated based on the Equation

% of tyrosinase activity = (Abs(S)/Abs(C)) × 100%(3)

where Abs(S)—the absorbance of the sample (extract + tyrosinase + substrate), and Abs(C)—the absorbance of the control sample (solvent + tyrosinase + substrate). Each sample was analyzed in three independent repetitions.

### 3.10. Murine Tyrosinase Inhibitory Assay

Murine tyrosinase inhibition by *Allium* sp. extracts was analysed using B16F10 murine melanoma cell lysate, as described by Uchida et al. with modifications [61]. B16F10 were cultured in DMEM high glucose supplemented with 10% FBS in humidified atmosphere at 37 °C and 5% CO_2_. 8 × 10^6^ cells were lysed in 5 mL phosphate buffer (100 mM, pH 6.8) containing 1% Triton X-100 in a sonicator ice cold water bath for one hour. Following centrifugation (10 min, 13 000 rpm), the supernatant was collected as murine tyrosinase solution and the protein concentration was measured using a DC Protein Assay (Bio-Rad, Warsaw, Poland). The volume of cell lysate containing 20 µg protein was mixed with 20 µL of *Allium* sp. extract, 40 µL 4 mM l-DOPA, and 100 mM phosphate buffer pH 6.8 (up to 200 µL). The absorbance at λ = 450 nm was measured following four hours of incubation at 37 °C. Kojic acid was used as a known tyrosinase inhibitor control. The activity of tyrosinase was calculated based on the Equation (3).

### 3.11. Statistical Analysis

The data were analyzed using GraphPad Prism 7.0 Software (GraphPad Software, San Diego, CA, USA). The statistical significance between results was assessed using one-way analysis of variance (ANOVA) followed by Tukey’s test. All data are showed as mean ± SD. The experiments were conducted in at least three repetitions.

## 4. Conclusions

The performed studies show a rich composition of the studied extracts of different polarity that were obtained from the bulbs and chives of *A. galanthum* and bulbs of *A. turkestanicum* growing in Kazakhstan. Flavonoids and their glucosides, organic acids, long-chain oxygenated saturated alkanes, fatty acids, ethyl esters of fatty acids and sterols were found in the tested plant material. The analyzed unpolar diethyl ether extracts were found to exhibit stronger biological activity in comparison with water and water-ethanol extracts of different ratios. The first extracts were found to be strong antimicrobial agents not only against the tested Gram-positive bacteria, but also inhibited the growth of several strains of Gram-negative bacteria and yeasts possibly due to the presence of 16-hentriacontanone (= palmitone) and 1-tritriacontanol among other constituents of the extracts. The presence of flavonoids, oxygenated long-chain alkanes and alkohols was certainly influencing an increased antiradical potential of the extracts. The highest potential was measured for diethyl ether extracts from chives of AG and bulbs of AT. The AG bulb diethyl ether extracts were also proved to inhibit murine tyrosinase until 54% of its activity, which can confirm its promising whitening properties.

The development of the cosmetics market, dictated by an increased awareness of consumers, breach the production of high-value cosmetics-devoid of toxic ingredients, based on recipes containing compounds of natural origin. In line with these trends, it is necessary to search for new cosmetic ingredients that will protect the skin against harmful, skin irritating and oxidative stress-inducing factors to improve its condition.

So far, only the extract from the bulbs of *A. cepa* is registered in the CosIng database (EU’s Iventory of Cosmetic Ingredients) as a safe cosmetic ingredient suitable for antimicrobial, anti-dandruff, anti-ageing, antioxidant, emollient and conditioning products. It is worth noting that a constantly growing number of scientific publications also confirm the same properties for other *Allium* spp., including the herein studied Kazakh species. The ability of onion extracts to inhibit the activity of the enzyme associated with skin discoloration - tyrosinase, confirmed by the above-described tests, is certainly of great value. The confirmed antiradical properties of the tested extracts when added to cosmetics could exhibit soothing properties against the negative impact of the environmental stressors that influence the skin function and results with an increased generation of reactive oxygen species. Finally, the antimicrobial potential of the tested samples will be important for preserving the cosmetic product, but also for inhibiting the development of bacterial infections on the surface of the skin exposed to irritation and acne.

To conclude, the herein described results of the conducted survey strongly support the application of *A. turkestanicum* and *A. galanthum* extracts in cosmetics.

## Figures and Tables

**Table 1:** The list of detected metabolites in the 50% ethanol extracts from different organs of Kazakhstani onions—*Allium* *galanthum* and *Allium* *turkestanicum* (AGb, AGb, ATb).

No.	Ion Species	Rt	Molecular Formula	*m*/*z* Calculated	*m*/*z* Experimental	Δppm	RDB	MS/MS Fragments	Proposed Compound	Ref.	AGb	AGc	ATb
**1**	[M−H]^−^	2.7	C_6_H_8_O_7_	191.0197	191.0209	−6.11	3	129, 111	**Citric acid**	[22]	+	+	+
**2**	[M−H]^−^	8.2	C_33_H_40_O_22_	787.1938	787.1986	−6.03	14	625, 463, 301	**Quercetin triglycoside**	[23]	+	+	−
**3**	[M−H]^−^	8.7	C_21_H_22_O_12_	465.1038	465.1067	−6.11	11	303, 285	**Taxifolin glucoside**	[24]	++	+	Tr
**4**	[M−H]^−^	9.15	C_7_H_12_O_5_	175.0612	175.0623	−6.26	2	157, 115	**Propylmalic acid**	[25]	+	+	+
**5**	[M−H]^−^	10.4	C_27_H_30_O_17_	625.1410	625.1463	−8.43	13	463, 301	**Quercetin dihexoside**	[23]	++	++	+
**6**	[M−H]^−^	10.5	C_27_H_30_O_16_	609.1461	609.1515	−8.84	13	446, 283	**Kaempferol diglucoside**	[23]	+	++	+
**7**	[M−H]^−^	11.6	C_27_H_30_O_16_	609.1461	609.1495	−5.56	13	446, 285	**Kaempferol diglucoside**	[23]	+	++	+
**8**	[M−H]^−^	13.1	C_21_H_21_O_11_^+^	449.1089	449.1109	−4.37	11	287	**Cyanidin glucoside**	[26]	+	Tr	Tr
**9**	[M−H]^−^	15.0	C_41_H_72_O_10_^+^	723.5053	723.5097	−6.11	6	677, 255	**Unknown 1**		++	+	++
**10**	[M−H]^−^	16.0	C_15_H_12_O_7_	303.0510	303.0481	9.62	10	285	**Dihydroquercetin**	[27]	+	+	Tr
**11**	[M−H]^−^	16.9	C_28_H_32_O_17_	639.1567	639.1573	−0.98	13	315	**Isorhamnetin diglucoside**	[26]	+	+	Tr
**12**	[M−H]^−^	18.4	C_55_H_80_O_6_	836.596	836.5945	1.84	15.5	790, 483	**Unknown 2**		++	+	++
**13**	[M−H]^−^	18.7	C_21_H_20_O_12_	463.0882	463.0907	−5.39	12	301, 151	**Isoquercetin**	[22]	++	++	+
**14**	[M−H]^−^	19.8	C_21_H_20_O_11_	447.0933	447.0894	8.67	12	284, 174	**Kaempferol glucoside**	[28]	+	+	−
**15**	[M−H]^−^	20.8	C_22_H_22_O_12_	477.1038	477.1041	−0.52	12	314	**Isorhamnetin glucoside**	[26]	++	++	Tr
**16**	[M−H]^−^	29.6	C_15_H_10_O_7_	301.0354	301.0381	−9.02	11	−	**Quercetin**	[22]	+	++	+
**17**	[M−H]^−^	35.5	C_15_H_10_O_6_	285.0405	285.0391	4.76	11	−	**Kaempferol**	[29]	+	++	-

(Ion—type of ionization, Rt—retention time in minutes, RDB—ring and double bond equivalent, Ref—references, Δppm—the error of measurement expressed in mmu, AGb—*Allium* *galanthum* bulbs, AGc—*Allium* *galanthum* chives, ATb—*Allium* *turkestanicum* bulbs, Tr—traces, + detected, − not detected. The intensity of the peaks is marked with the number of pluses, namely ++ the peaks with 10^5^ intensity and + with 10^4^ intensity).

**Table 2:** The list of compounds identified in diethyl ether extracts of Kazakhstani onions—*Allium* *galanthum* and *Allium* *turkestanicum* after their analysis by GC-MS method.

No.	Compound Name	Molecular Formula	RI*	AGb	AGc	ATb
1	Nonanal	C_9_H_18_O	1106	+		
2	Neophytadiene (isomer II)	C_20_H_38_	1836		+	
3	Hexadecanoic acid	C_16_H_32_O_2_	1968	+	+	+
4	Hexadecanoic acid ethyl ester	C_18_H_36_O_2_	1992	+		
5	3,13-Octadecadien-1-ol	C_18_H_34_O	2094	+		+
6	Linoleic acid	C_18_H_32_O_2_	2146			++
7	Linoleic acid ethyl ester	C_20_H_36_O_2_	2162	+		
8	Oleic acid ethyl ester	C_20_H_38_O_2_	2168	+		
9	Tetracosane	C_24_H_50_	2402	+	+	+
10	1-Tetracosanol	C_24_H_50_O	2475	+		
11	Pentacosane	C_25_H_52_	2502	+	+	+
12	Hexacosane	C_26_H_54_	2603	+	+	+
13	1-Hexacosanol	C_26_H_54_O	2681	++	+	
14	Heptacosane	C_27_H_56_	2703	+	+	+
15	Tetracosanoic acid ethyl ester	C_26_H_52_O_2_	2798	+		
16	Octacosane	C_28_H_58_	2801		+	
17	Squalene	C_30_H_50_	2815	+	+	+
18	1-Octacosanol	C_28_H_58_O	2882	+++	+	+
19	Nonacosane	C_29_H_60_	2904	+	+	+
20	Triacontane	C_30_H_62_	3003	+	+	+
21	1-Triacontanol	C_30_H_62_O	3084	+++	++	+
22	Hentriacontane	C_31_H_64_	3105	+++	+++	+++
23	Cholesterol	C_27_H_46_O	3147	+	+	
24	Dotriacontane	C_32_H_66_	3204	+	+	
25	Lathosterol	C_27_H_46_O	3210	+	+	
26	14-Methylergost-8-en-3-ol	C_29_H_50_O	3226	+	+	+
27	1,30-Triacontanediol	C_30_H_62_O_2_	3251		+	
28	1-Dotriacontanol	C_32_H_66_O	3285	++	+	
29	16-Hentriacontanone	C_31_H_62_O	3293		+++	+
30	Tritriacontane	C_33_H_68_	3305	++	+	+
31	1-Tritriacontanol	C_33_H_68_O	3382		+++	

RI*—retention index relative to n-alkane series; the relative content of identified compounds was indicated as high (+++), moderate (++) or low (+) based on the peak area.

**Table 3:** MIC (mg/mL) and MBC (mg/mL) and MFC (mg/mL) values of dried ether extracts obtained from two Kazakh species of onions-*Allium galanthum* and *Allium* *turkestanicum* against the reference strains.

Microbial Strains	Extracts					
	AGb		AGc		ATb	
Gram-positive bacteria	MIC	MBC	MIC	MBC	MIC	MBC
*Staphylococcus aureus* ATCC 25923	1	2	0.5	1	0.5	1
*Staphylococcus aureus* ATCC 29213	2	8	1	2	1	2
*Staphylococcus aureus* ATCC BAA1707	2	2	1	1	1	1
*Staphylococcus epidermidis* ATCC 12228	1	4	0.5	1	0.5	1
*Bacillus subtilis* ATCC 6633	0.25	1	0.125	0.5	0.125	0.5
*Bacillus cereus* ATCC 10876	0.25	2	0.25	2	0.25	2
*Micrococcus luteus* ATCC 10240	1	4	0.5	1	0.5	1
Gram-negative bacteria	MIC	MBC	MIC	MBC	MIC	MBC
*Salmonella typhimurium* ATCC 14028	4	8	1	8	1	8
*Bordetella bronchiseptica* ATCC 4617	1	8	1	8	1	8
*Klebsiella pneumoniae* ATCC 13883	4	16	1	8	1	8
*Proteus mirabilis* ATCC 12453	4	16	1	8	1	8
*Escherichia coli* ATCC 25922	2	8	1	4	1	4
Yeasts	MIC	MFC	MIC	MFC	MIC	MFC
*Candida parapsilosis* ATCC 22019	1	2	0.5	0.5	0.5	0.5
*Candida albicans* ATCC 2091	1	1	0.25	0.25	0.25	0.25
*Candida albicans* ATCC 10231	0.125	0.5	0.125	0.5	0.125	0.5
*Candida glabrata* ATCC 90030	0.25	0.25	0.25	0.25	0.25	0.25
*Candida krusei* ATCC 14243	0.25	1	0.125	0.25	0.125	0.25

**Table 4:** MIC (mg/mL) and MBC (mg/mL) and MFC (mg/mL) values of different dried extracts obtained from *Allium* *galanthum* against the reference strains.

Microbial Strains	Extracts							
	AGb_H_2_O		AGb_Et50%		AGb_Et70%		AGb_Et96%	
Gram-positive bacteria	MIC	MBC	MIC	MBC	MIC	MBC	MIC	MBC
*Staphylococcus aureus* ATCC 25923	16	32	16	32	16	32	16	32
*Staphylococcus aureus* ATCC 29213	32	32	32	32	16	32	16	32
*Staphylococcus aureus* ATCC AA1707	32	32	32	32	16	32	16	32
*Staphylococcus epidermidis* ATCC 12228	32	32	32	32	16	32	16	32
*Bacillus subtilis* ATCC 6633	16	16	16	16	16	16	16	16
*Bacillus cereus* ATCC 10876	16	32	16	32	16	32	16	32
*Micrococcus luteus* ATCC 10240	32	32	32	32	16	32	16	32
Gram-negative bacteria	MIC	MBC	MIC	MBC	MIC	MBC	MIC	MBC
*Salmonella typhimurium* ATCC 14028	16	32	16	32	16	32	16	32
*Bordetella bronchiseptica* ATCC 4617	16	16	16	16	16	16	16	16
*Klebsiella pneumoniae* ATCC 13883	16	32	16	32	16	32	16	32
*Proteus mirabilis* ATCC 12453	16	16	16	16	16	16	16	16
*Escherichia coli* ATCC 25922	16	32	16	32	16	32	16	32
Yeasts	MIC	MFC	MIC	MFC	MIC	MFC	MIC	MFC
*Candida parapsilosis* ATCC 22019	8	8	8	8	8	8	8	8
*Candida albicans* ATCC 2091	8	8	8	8	8	8	8	8
*Candida albicans* ATCC 10231	8	8	8	8	8	8	8	8
*Candida glabrata* ATCC 90030	8	16	8	16	8	16	8	16
*Candida krusei* ATCC 14243	8	16	8	16	8	16	8	16

Legend: AGb_H_2_O, AGb_Et50%, AGb_Et70%, and AGb_Et96% are the water, 50% ethanol, 70% ethanol and 96% ethanol extracts, respectively.

**Table 5:** MIC (mg/mL) and MBC (mg/mL) and MFC (mg/mL) values of different dried extracts obtained from *Allium* *turkestanicum* against reference strains.

Microbial Strains	Extracts							
	ATb_H_2_O		ATb_Et50%		ATb_Et70%		ATb_Et96%	
Gram-positive bacteria	MIC	MBC	MIC	MBC	MIC	MBC	MIC	MBC
*Staphylococcus aureus* ATCC 25923	4	16	2	16	2	16	2	16
*Staphylococcus aureus* ATCC 29213	16	16	8	16	4	8	4	8
*Staphylococcus aureus* ATCC AA1707	8	8	4	16	4	8	2	4
*Staphylococcus epidermidis* ATCC 12228	8	8	4	8	4	8	2	4
*Bacillus subtilis* ATCC 6633	8	16	4	8	4	8	4	4
*Bacillus cereus* ATCC 10876	8	16	4	16	4	16	4	16
*Micrococcus luteus* ATCC 10240	8	8	4	8	4	8	2	4
Gram-negative bacteria	MIC	MBC	MIC	MBC	MIC	MBC	MIC	MBC
*Salmonella typhimurium* ATCC 14028	8	8	8	8	4	8	4	4
*Bordetella bronchiseptica* ATCC 4617	8	8	4	4	4	4	2	4
*Klebsiella pneumoniae* ATCC 13883	8	8	4	4	4	4	4	4
*Proteus mirabilis* ATCC 12453	8	8	4	4	4	4	4	4
*Escherichia coli* ATCC 25922	8	8	4	4	4	4	4	4
Yeasts	MIC	MFC	MIC	MFC	MIC	MFC	MIC	MFC
*Candida parapsilosis* ATCC 22019	1	2	1	1	0.5	1	0.5	1
*Candida albicans* ATCC 2091	1	2	0.5	1	0.5	1	0.5	1
*Candida albicans* ATCC 10231	1	2	0.5	1	0.5	1	0.5	1
*Candida glabrata* ATCC 90030	2	4	1	2	1	2	1	2
*Candida krusei* ATCC 14243	2	2	1	2	1	2	1	2

Legend: ATb_H_2_O, ATb_Et50%, ATb_Et70%, and ATb_Et96% are the water, 50% ethanol, 70% ethanol and 96% ethanol extracts, respectively.

**Table 6:** The antioxidant activity of extracts from of Kazakhstani bulbs—*Allium* *galanthum* bulbs and chives (AGb and AGc, respectively) and *Allium* *turkestanicum* bulbs (ATb).

Sample	DPPH Assay	ABTS Assay	Sample	DPPH Assay	ABTS Assay
**AGc_H_2_O**	1256.72 ± 19.28	5.08 ± 0.07	**AGb_H_2_O**	1132.60 ± 23.01	2.23 ± 0.03
**AGc_Et96%**	1172.78 ± 10.15	3.70 ± 0.07	**AGb_Et96%**	1208.48 ± 21.39	4.76 ± 0.06
**AGc_Et50%**	1243.51 ± 24.49	4.87 ± 0.16	**AGb_Et50%**	1130.14 ± 17.27	2.54 ± 0.03
**AGc_Et70%**	1280.78 ± 14.72	4.53 ± 0.23	**AGb_Et70%**	1081.01 ± 46.66	2.16 ± 0.03
Sample	DPPH Assay	ABTS Assay	Sample	DPPH Assay	ABTS Assay
**ATb_H_2_O**	1020.35 ± 40.30	0.61 ± 0.03	**ATb_Eth**	1501.14 ± 10.95	50.85 ± 2.90
			**AGc_Eth**	975.92 ± 35.94	35.68 ± 0.58
**ATb_Et96%**	1071.04 ± 17.82	1.81 ± 0.04	**AGb_Eth**	19,274.78 ± 92.11	37.51 ± 2.31
**ATb_Et70%**	1151.29 ± 12.85	2.65 ± 0.01	**Vit C**	1535.46 ± 8.88	1252.80 ± 8.02

The results are displayed as mg Trolox equivalents per g of dried extract.

## Data Availability

The data supporting the results are present in the manuscript and in the Supplementary Materials.

## Supplementary Materials

The following are available online, Figure S1: Mass chromatograms recorded for 50% ethanol extracts of Kazakhstani bulbs (top: *Allium* *galanthum* onion extract in negative ion mode, in positive ion mode, *Allium* *galanthum* chives extract in negative ion mode, in positive ion mode, *Allium* *turkestanicum* onion extract in positive ion mode, in negative ion mode); Table S1: The MS/MS fragmentation spectra of the identified compounds; Figure S2: Scavenging of DPPH free radical by *A. galanthus* and *A. turkestanicum* extracts; graphs show mean ± SD (*n* = 3); Figure S3. Scavenging of ABTS free radical by *A. galanthus* and *A. turkestanicum* extracts; graphs show mean ± SD (*n* = 3); Table S2. The list of screened compounds during the compositional studies.

## Sample Availability

Samples of the plant material are available from the authors.
