# Supplementary material for: The Phenolic Compounds Profile and Cosmeceutical Significance of Two Kazakh Species of Onions: Allium galanthum and A. turkestanicum
Source: Molecules. 2021 Sep 9;26(18):5491. doi: 10.3390/molecules26185491 (PMC8467033; doi:10.3390/molecules26185491)
Supplement: Supplementary file 1 [file molecules-26-05491-s001.zip › molecules-1363935-supplementary.pdf]

molecules

MDPI

Supplementary file

The phenolic compounds profile and cosmeceutical significance of two Kazakh species of onions: *Allium galanthum* and *A. turkestanicum*

Gulnara Kadyrbayeva [1], Justyna Zagórska [2], Agnieszka Grzegorczyk [3], Katarzyna Gaweł-Bęben [4], Marcelina Strzępek-Gomółka [4], Agnieszka Ludwiczuk [5], Karolina Czech [4], Manoj Kumar [6], Wojciech Koch [2], Anna Malm [3], Kazimierz Głowniak [4], Zuriyadda Sakipova [1] and Wirginia Kukula-Koch [7,*]

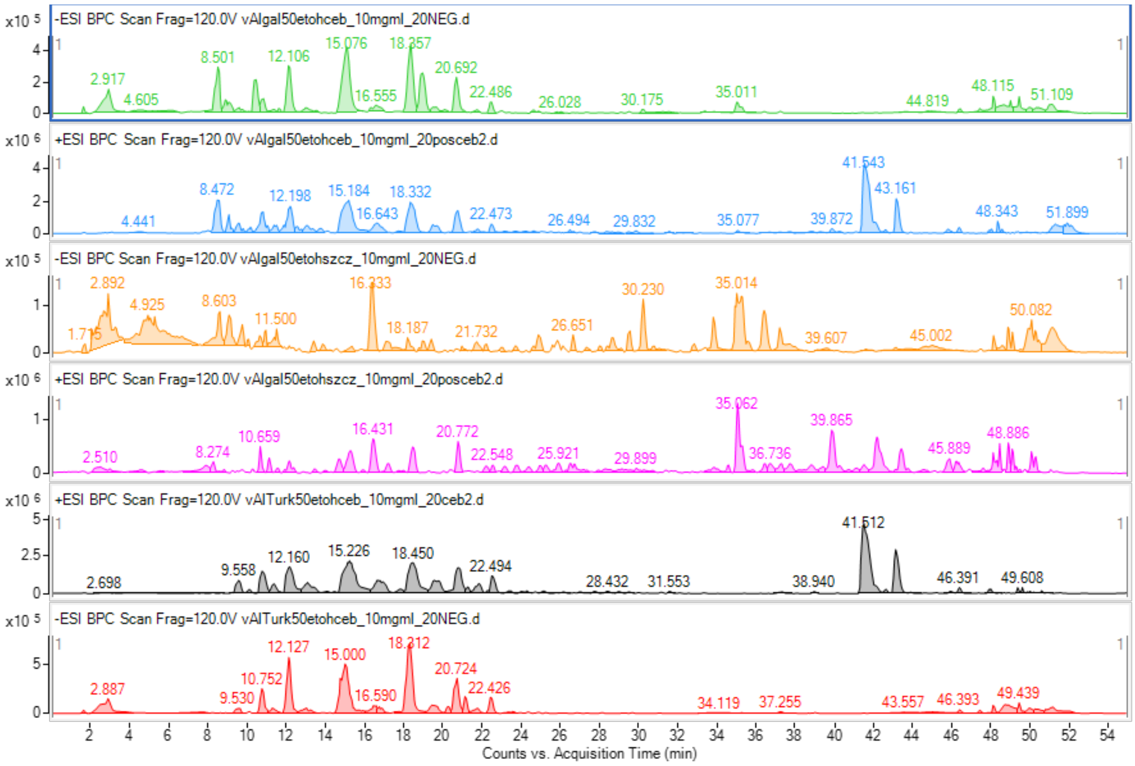

Figure S1. Mass chromatograms recorded for 50% ethanol extracts of Kazakhstani bulbs (top: *Allium galanthum* onion extract in negative ion mode, in positive ion mode, *Allium galanthum* chives extract in negative ion mode, in positive ion mode, *Allium turkestanicum* onion extract in positive ion mode, in negative ion mode).

Table S1. The MS/MS fragmentation spectra of the identified compounds.

Isoquercetin

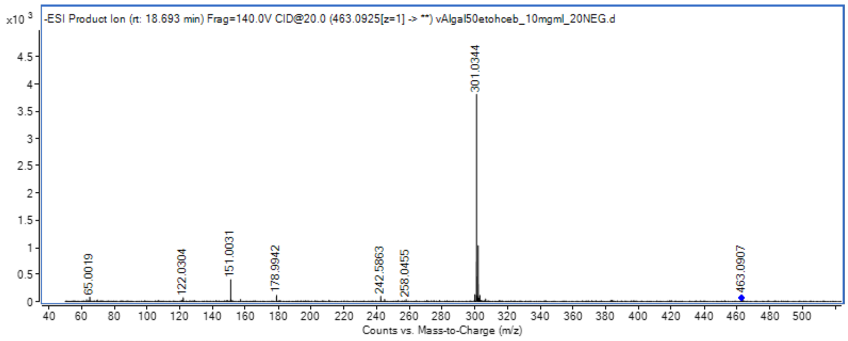

Quercetin dihexoside

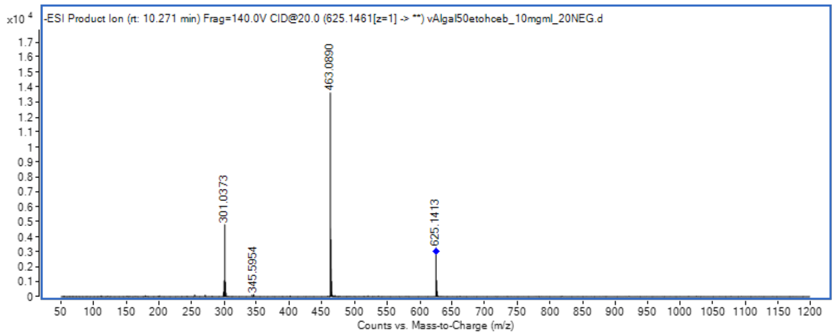

Kaempferol

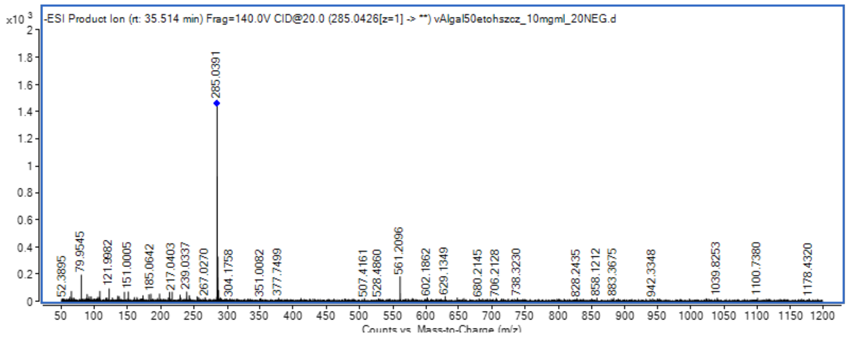

Kaempferol diglucosides

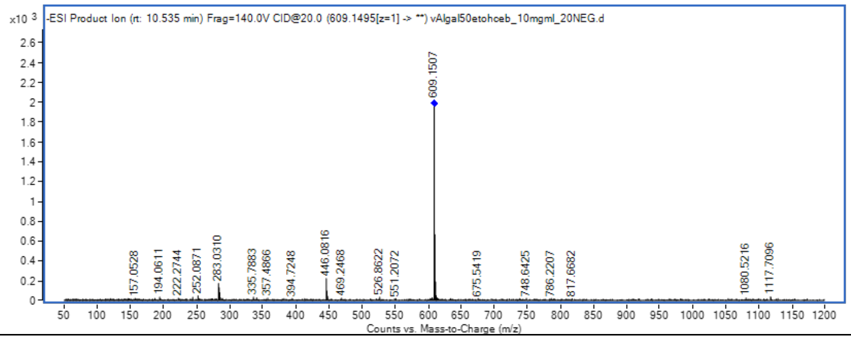

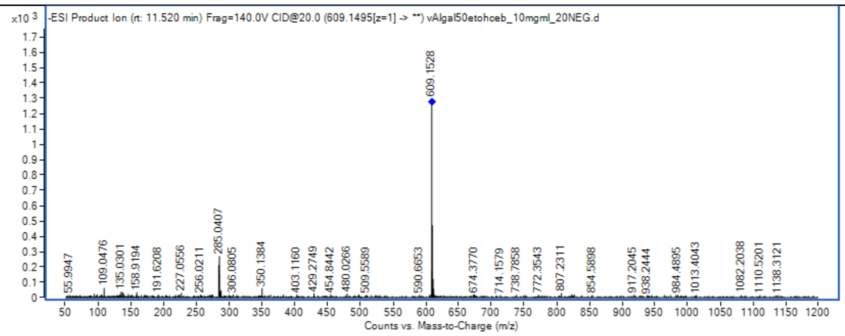

Kaempferol glucoside

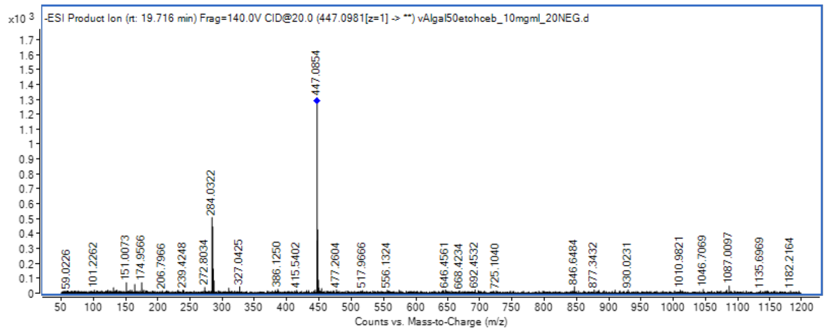

Isorhamnetin diglucoside

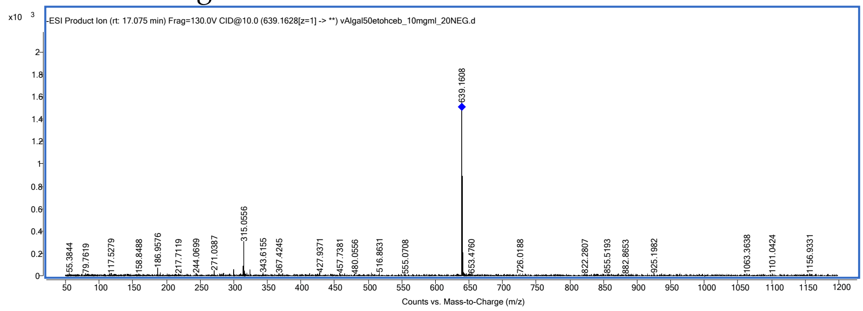

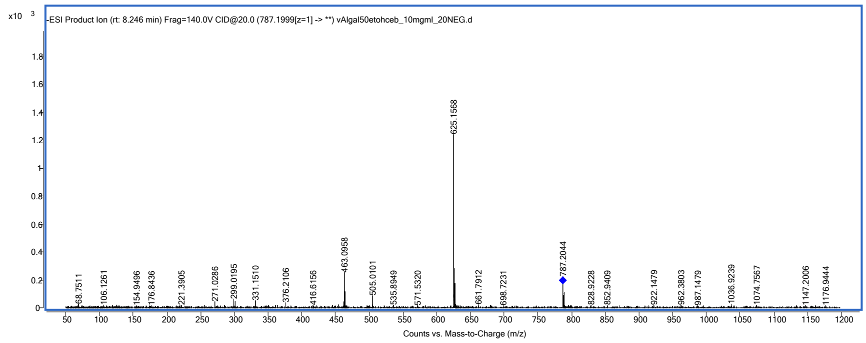

Quercetin triglycoside

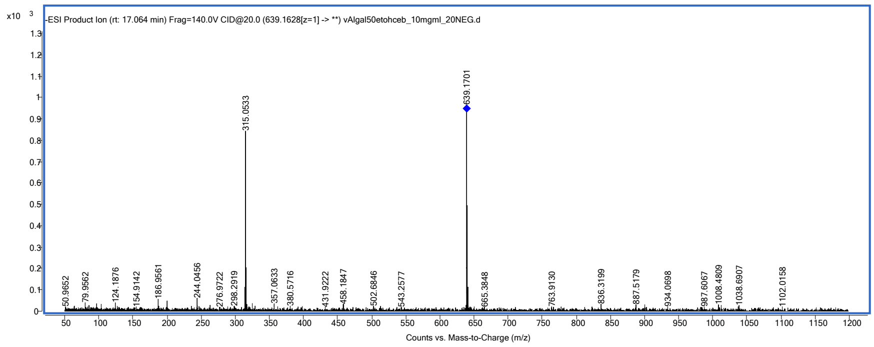

Isorhamnetin glucoside

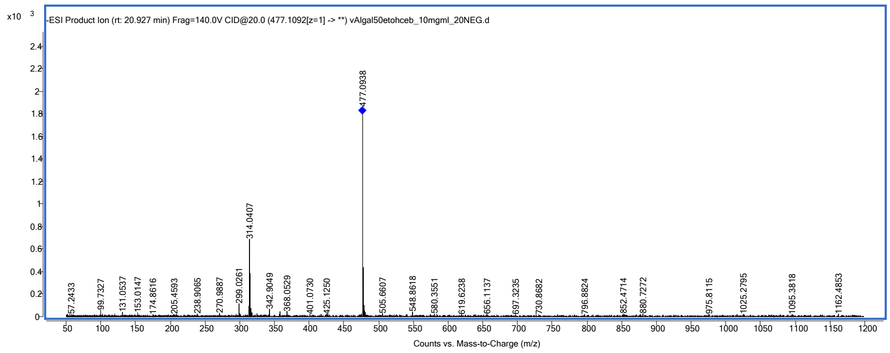

Cyanidin glucoside

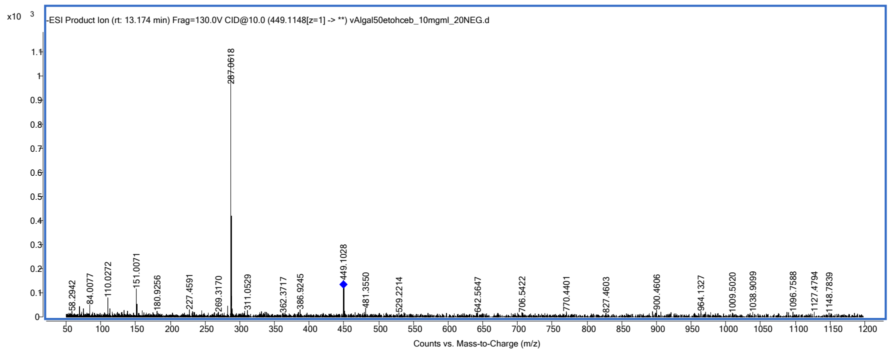

Unknown 1

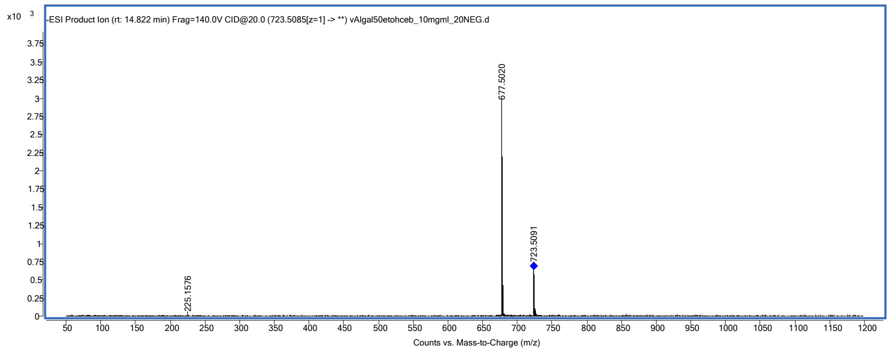

Unknown 2

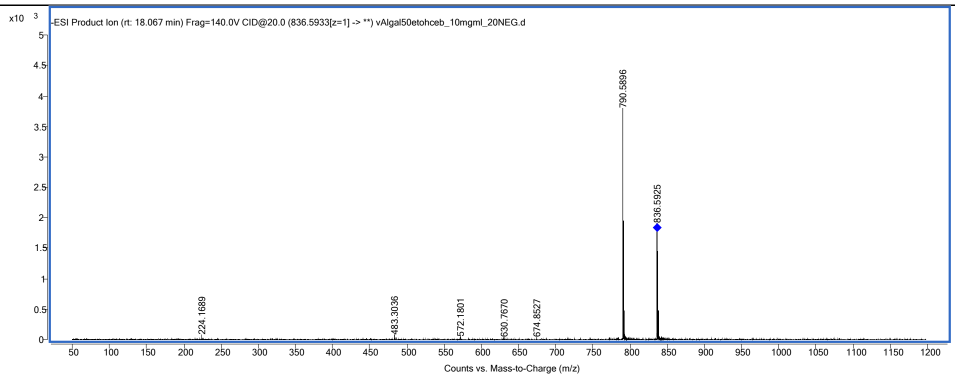

Taxifolin glucoside

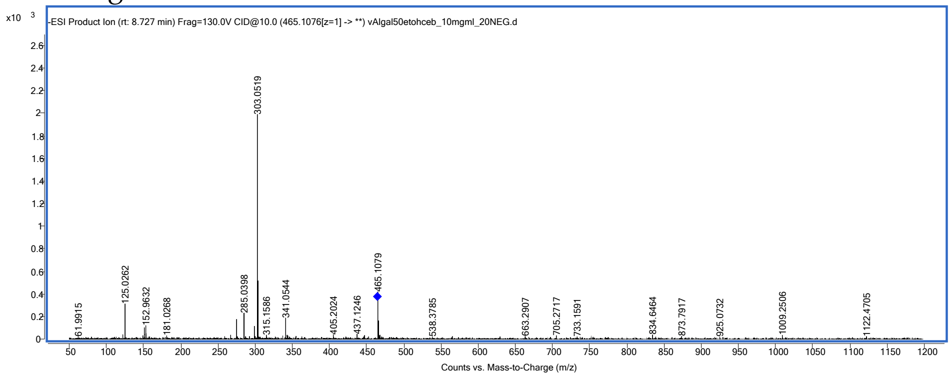

Propylmalic acid

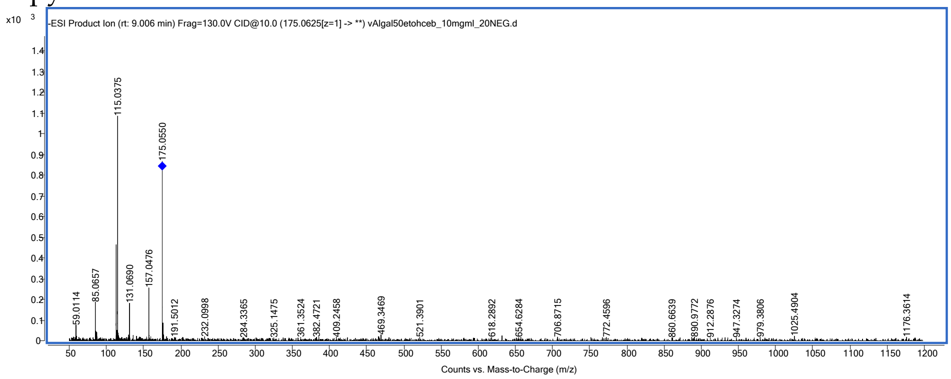

Dihydroquercetin

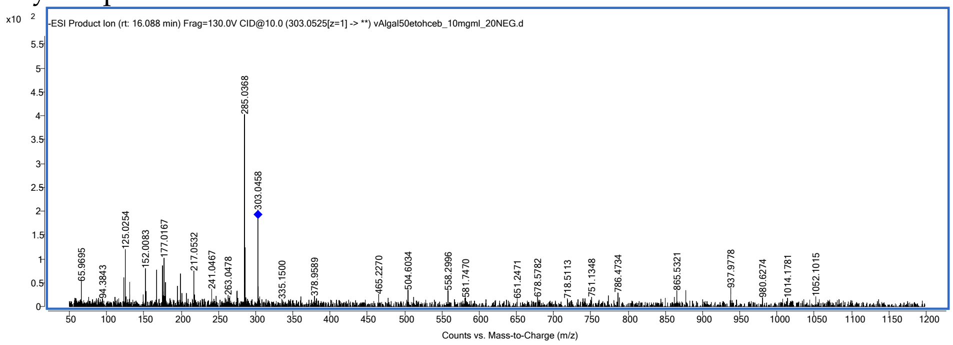

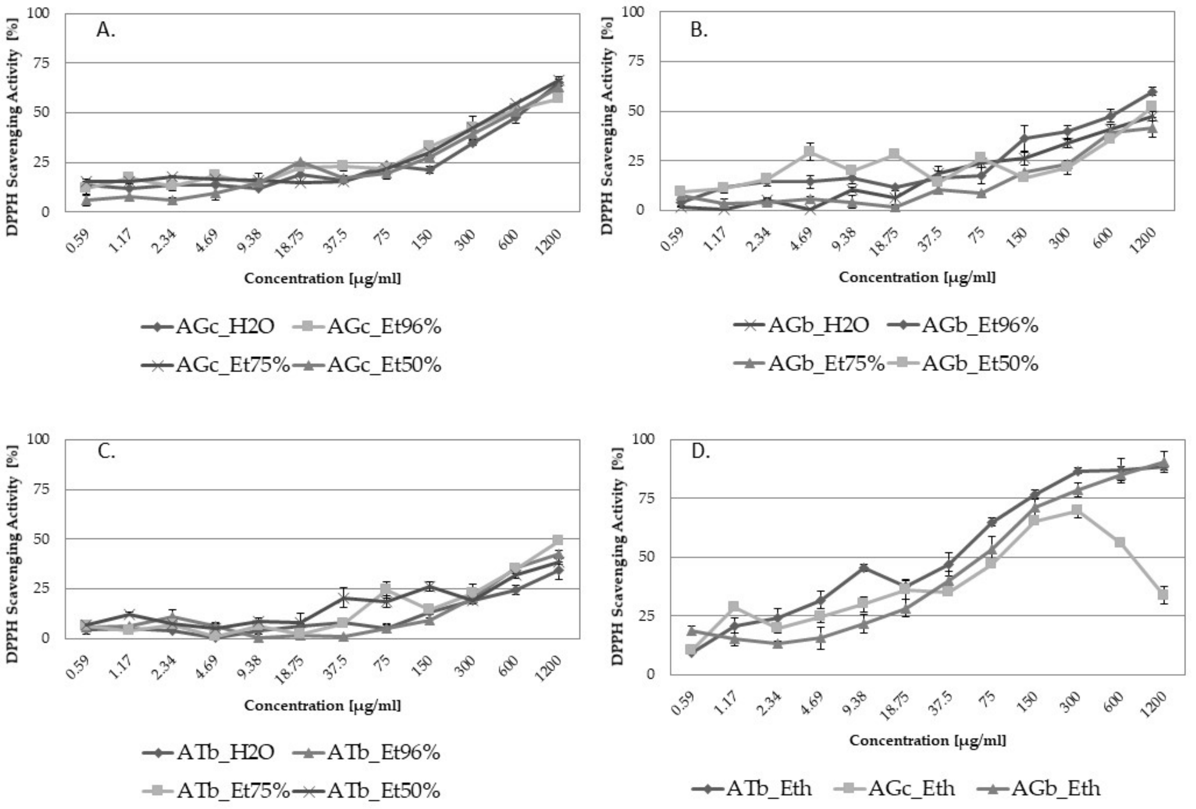

Figure S2. Scaveging of DPPH free radical by *A. galanthus* and *A. turkestanicum* extracts; graphs show mean ±SD ($n = 3$)

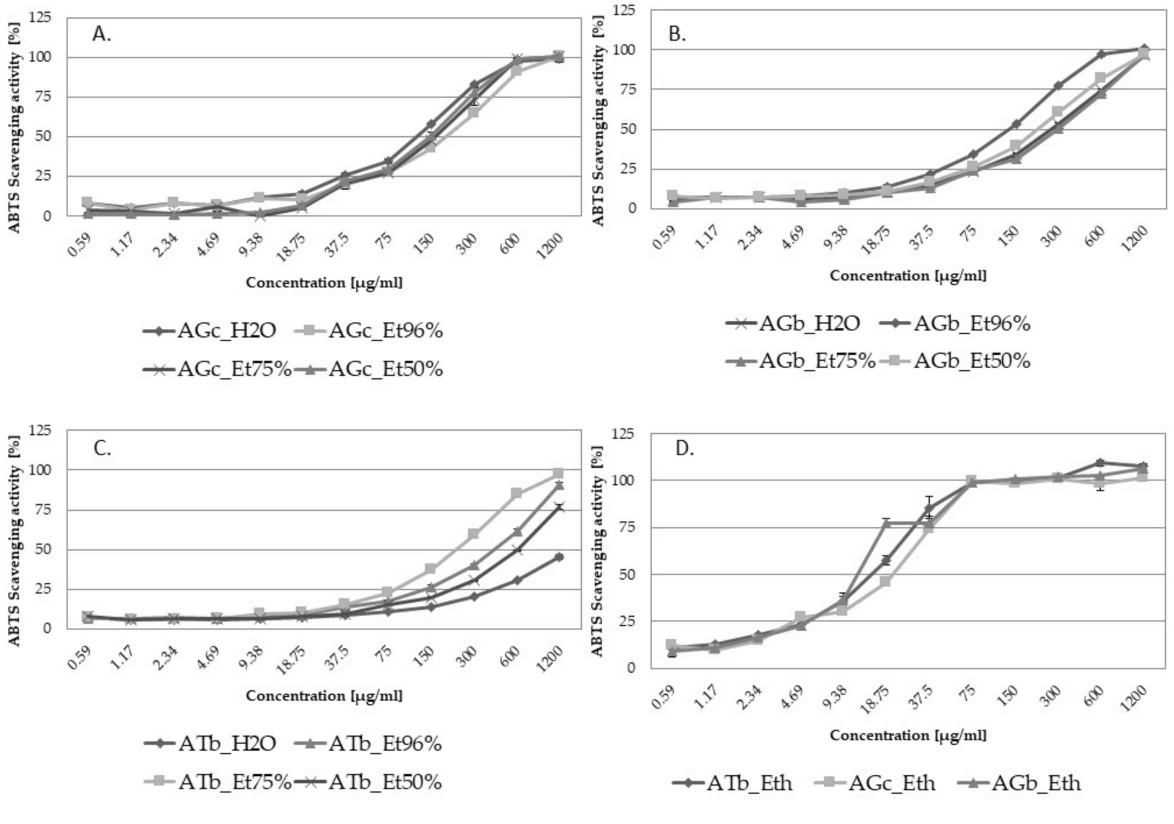

Figure S3. Scaveging of ABTS free radical by *A. galanthus* and *A. turkestanicum* extracts; graphs show mean ±SD (n = 3)

Table S2. The list of screened compounds during the compositional studies

			m/z calculated		
	Ferulic acid		194.057910<br>193.050085<br>195.065735		

			224.068475<br>223.060650<br>225.076300		

			286.047740<br>285.039915<br>287.055565		
	Kaempferol		286.047740<br>285.039915<br>287.055565		

		[M+H]+ $C_{15}H_{11}O_6$			
			270.052825<br>269.052825<br>271.060650		

			177.045966<br>176.038141<br>178.053791		

			162.017309<br>161.009484<br>163.025133		
		$C_{29}H_{50}O$	414.386165<br>413.378340<br>415.393990		

			380.214015<br>379.206190<br>381.221840		

			124,052430<br>123.044605<br>125.060255		
			72.021130<br>71.013305<br>73.028955		

			892.535316<br>891.527491<br>893.543141		

		C55H70O6N4Mg<br>[M-H]- C55H69O6N4Mg<br>[M+H]+ C55H71O6N4Mg	906,514581<br>905.506756<br>907,522406		
		$C_6H_8O_6$<br>[M-H]- $C_6H_7O_6$	176,032090<br>175.024265<br>177,039915		
			177.045966<br>176.038141<br>178.053791		

		$C_5H_{11}NO_2S$<br>[M+H]+ $C_5H_{12}NO_2S$	149.051051<br>148,043226<br>150,058876		
		$C_9H_{14}OS_3$<br>[M-H]- $C_9H_{13}OS_3$<br>[M+H]+ $C_9H_{15}OS_3$	234.020681<br>233.012856<br>235.028506		
		$C_9H_{14}OS_3$<br>[M-H]- $C_9H_{13}OS_3$<br>[M+H]+ $C_9H_{15}OS_3$	234.020681<br>233.012856<br>235.028506		

		$C_6H_8S_2$<br>[M-H]- $C_6H_7S_2$<br>[M+H]+ $C_6H_9S_2$	144.006744<br>142.998919<br>145.014569		
		$C_6H_{10}S$<br>[M-H]- C_6H_9S<br>[M+H]+ $C_6H_{11}S$	114.050322<br>113.042497<br>115.058146		
		$C_6H_{10}S_2$<br>[M-H]- $C_6H_9S_2$<br>[M+H]+ $C_6H_{11}S_2$	146.022394<br>145.014569<br>147.030219		
		$C_6H_{10}S_3$<br>[M-H]- $C_6H_9S_3$	177.994466<br>176.986641		

		[M+H]+ $C_6H_{11}S_3$	179.002291		
		C_4H_8S<br>[M-H]- C_4H_7S<br>[M+H]+ C_4H_9S	88.034672<br>87.026847<br>89.042497		
		$C_{69}H_{114}O_{39}$<br>[M-H]- $C_{69}H_{113}O_{39}$<br>[M+H]+ $C_{69}H_{115}0_{39}$	1566.693735<br>1565.685910<br>1567.701560		5
		$C_{62}H_{100}O_{34}$<br>[M-H]- $C_{62}H_{99}O_{34}$<br>[M+H]+ $C_{62}H_{101}O_{34}$	1388.609610<br>1387.601785<br>1389.617435		

		$C_{62}H_{102}O_{36}$<br>[M-H]- $C_{62}H_{101}O_{36}$<br>[M+H]+ $C_{62}H_{103}O_{36}$	1422.615090<br>1421.607265<br>1423.622915		
		$C_{56}H_{94}0_{27}$<br>[M-H]- $C_{56}H_{93}0_{27}$<br>[M+H]+ $C_{56}H_{95}0_{27}$	1198.598254<br>1197.590430<br>1199.606080		

		$C_{62}H_{104}O_{32}$<br>[M-H]- $C_{62}H_{103}O_{32}$<br>[M+H]+ $C_{62}H_{105}O_{32}$	1360.651080<br>1359.643255<br>1361.658905		
		-	-		
		$C_5H_{11}NO_3S_2$<br>[M-H]- $C_5H_{10}NO_3S_2$<br>[M+H]+ $C_5H_{12}NO_3S_2$	197.018038<br>196.010213<br>198.025863		

		$C_{10}H_{18}N_2O_6S_2$<br>[M-H]- $C_{10}H_{17}N_2O_6S_2$<br>[M+H]+ $C_{10}H_{19}N_2O_6S_2$	326.060632<br>325.052807<br>327.068456		
		$C_4H_9NO_3S$<br>[M-H]- $C_4H_8NO_3S$<br>[M+H]+ $C_4H_{10}NO_3S$	151.030316<br>150.022491<br>152.038141		

		$C_6H_{13}NO_3S$<br>[M-H]- $C_6H_{12}NO_3S$<br>[M+H]+ $C_6H_{14}NO_3S$	179.061616<br>178.053791<br>180.069440		
		$C_7H_{10}N_20_3S$<br>[M-H]- $C_7H_9N_20_3S$<br>[M+H]+ $C_7H_{11}N_20_3S$	202.041215<br>201.033390<br>203.049040		

		$C_8H_{10}N_2O_3S$<br>[M-H]- $C_8H_9N_2O_3S$<br>[M+H]+ $C_8H_{11}N_2O_3S$	214.041215<br>213.033390<br>215.049040		
		$C_8H_{10}N_2O_3S$<br>[M-H]- $C_8H_9N_2O_3S$<br>[M+H]+ $C_8H_{11}N_2O_3S$	214.041215<br>213.033390<br>215.049040		

		$C_{18}H_{36}O$<br>[M-H]- $C_{18}H_{35}O$<br>[M+H]+ $C_{18}H_{37}O$	268.276615<br>267.268789<br>269.284440		
		$C_{2}H_{6}O$<br>[M-H]- $C_{2}H_{5}O$<br>[M+H]+ $C_{2}H_{7}O$	46.041865<br>45.034040<br>47.049690		
		$C_{29}H_{50}O$<br>[M-H]- $C_{29}H_{49}O$<br>[M+H]+ $C_{29}H_{51}O$	414.386165<br>413.378340<br>415.393990		

		$C_{29}H_{46}$<br>[M-H]- $C_{29}H_{45}$<br>[M+H]+ $C_{29}H_{47}$	394.359950<br>393.352125<br>395.367775		
		$C_{19}H_{40}O$<br>[M-H]- $C_{19}H_{39}O$<br>[M+H]+ $C_{19}H_{41}O$	284.307915<br>283.300090<br>285.315740		
		$C_{15}H_{32}O$<br>[M-H]- $C_{15}H_{31}O$<br>[M+H]+ $C_{15}H_{33}O$	228.245315<br>227.237490<br>229.253140		

		$C_6H_8S_2$<br>[M-H]- $C_6H_7S_2$<br>[M+H]+ $C_6H_9S_2$	144.006744<br>142.998919<br>145.014569		
		$C_{21}H_{20}O_{11}$<br>[M-H]- $C_{21}H_{19}O_{11}$<br>[M+H]+ $C_{21}H_{21}O_{11}$	448.100564<br>447.092740<br>449.108390		

		$C_{21}H_{20}O_{12}$<br>[M-H]- $C_{21}H_{19}O_{12}$<br>[M+H]+ $C_{21}H_{21}O_{12}$	464.095480<br>463.087655<br>465.103305		

		Kaempferol 3-O-glucoside-7-O-glucuronide<br>$C_{27}H_{28}O_{17}$<br>[M-H]- $C_{27}H_{27}O_{17}$<br>[M+H]+ $C_{27}H_{29}O_{17}$	624.132655<br>623.124830<br>625.140480		

		Kaempferol 3-neohesperidoside<br>$C_{27}H_{30}O_{15}$<br>[M-H]- $C_{27}H_{29}O_{15}$<br>[M+H]+ $C_{27}H_{31}O_{15}$	594.158475<br>593.150650<br>595.166300		
		$C_{27}H_{30}O_{16}$<br>[M-H]- $C_{27}H_{29}O_{16}$<br>[M+H]+ $C_{27}H_{31}O_{16}$	610.153390<br>609.145565<br>611.161215		

		Quercetin 3-glucoside-7-glucuronide<br>$C_{27}H_{28}O_{18}$<br>[M-H]- $C_{27}H_{27}O_{18}$<br>[M+H]+ $C_{27}H_{29}O_{18}$	640.127570<br>639.119745<br>641.135395		
		quercetin 3,7-di-O-β-D-glucoside<br>Quercetin 3,7-diglucoside<br>$C_{27}H_{30}O_{17}$<br>[M-H]- $C_{27}H_{29}O_{17}$<br>[M+H]+ $C_{27}H_{31}O_{17}$	626.148305<br>625.140480<br>627.156130		

		$C_9H_8O_3$<br>[M-H]- $C_9H_7O_3$<br>[M+H]+ $C_9H_9O_3$	164.047345<br>163.039520<br>165.055170		
		$C_{27}H_{30}O_{16}$<br>[M-H]- $C_{27}H_{29}O_{16}$<br>[M+H]+ $C_{27}H_{31}O_{16}$	610.153390<br>609.145565<br>611.161215		

([1] Laurian Vlase, Marcel Parvu, Elena Alina Parvu, and Anca Toiu. Chemical Constituents of Three Allium Species from Romania, 2012; [2] D.I.Stajner, A.Kapor, Exploring Allium species as a Source of Potential Medicinal Agents, 2006; [3] Kusterer, J., Fritsch, R. M., & Keusgen, M. (2011). Allium Species from Central and Southwest Asia Are Rich Sources of Marasmin. Journal of Agricultural and Food Chemistry, 59(15), 8289–8297.; [4] Modern state of the study of some plants of the genus Allium L.: a review article [5] Kuroda, M., Ori, K., Takayama, H., Sakagami, H., & Mimaki, Y. (2015). Karataviosides G–K, five new bisdesmosidic steroidal glycosides from the bulbs of Allium karataviense. Steroids, 93, 96–104. doi:10.1016/j.steroids.2014.09.010; [6] de Guzman Alvindia, D., Mangoba, M.A.A. Bioactivities of *Allium longicuspis* Regel against anthracnose of mango caused by *Colletotrichum gloeosporioides* (Penz.). Scientific Reports volume 10, Article number: 11367 (2020), [7] D. N. Olennikov. Flavonol glycosides from leaves of Allium microdictyon. Chemistry of Natural Compounds, Vol. 56, No. 6, November, 2020. 56(6):1035-1039)
